# Lysosomal Diseases and Neuropsychiatry: Opportunities to Rebalance the Mind

**DOI:** 10.3389/fmolb.2020.00177

**Published:** 2020-08-26

**Authors:** Timothy M. Cox

**Affiliations:** Department of Medicine, University of Cambridge, Cambridge, United Kingdom

**Keywords:** psychiatric manifestations, schizophrenia, lysosomal diseases, sphingolipids, late-onset Tay-Sachs disease, GM2 gangliosidoses, Thudichum, substrate-reduction therapy

## Abstract

The brain is the physical organ of the mind but efforts to understand mental illness within a neurobiological context have hitherto been unavailing. Mental disorders (anxiety, depression, bipolar disorder, and schizophrenia) affect about one fifth of the population and present an almost endless societal challenge at the frontier of human sciences. Prodigious technological advances in functional neuroimaging and large-scale genetics have not yet delivered the prospect of refined molecular understanding of mental illness beyond early anatomical descriptions of brain metabolism. However, intensive clinical phenotyping and quantitative metabolic studies using sophisticated radio-ligands in positron-emission tomography, persistently favor the neurobiological approach. This *Perspective* pursues a familiar maxim in Medicine, aptly summarized in the words of Arthur Koestler: “Nature is generous in her senseless experiments on mankind.” Hitherto, studies in neuropsychiatry have largely ignored rare genetic disorders but derangements of specific components within the cerebral laboratory offer rich opportunities for mechanistic exploration. Aberrant psychic behavior is characteristic of many inborn errors of metabolism and although each disorder represents a universe of its own, we are at a threshold for understanding, since contemporary genetics and cell biology furnish abundant materials to take on the perturbing enigma of mental derangement. A further development relates to orphan drugs with actions on defined molecular targets: these represent new ways to study the pathogenesis of psychiatric phenomena associated with rare diseases and in a manner not formerly possible. Here we introduce the frontier of schizophrenia and its strong association with late-onset Tay-Sachs disease as a paradigm to explore.

## Introduction

The nature of the mind and mental aberrations are unclaimed frontiers of medical science. Despite twin studies indicating up to 80% heritability (Cardno et al., 2012) and intensive genetic studies of common traits such as autism, schizophrenia and bipolar disorder, psychotherapeutic innovation is stagnant. Links between genetic variants and mental health may inform rational drug design, but since key interactions between heredity and the psychological environment are currently beyond our understanding (Misiak et al., 2018), the prospects for better treatments are poor.

The brain is frequently affected by metabolic disorders and about 2/3 of patients with lysosomal diseases have neurodegenerative complications (Walterfang et al., 2013; Sharma et al., 2018; Platt et al., 2019). Given the central rôle of lysosomes in autophagy, energy-sensing and membrane dynamics in metabolically active but long-lived neural cells (Sharma et al., 2018; Ballabio and Bonifacino, 2020), florid mental disturbances–in some cases indistinguishable from classical schizophrenia–may be presenting features (Walterfang et al., 2013). The unique biology of the lysosome offers spectacular opportunities for therapeutic complementation and this, combined with incentives of the orphan drug legislation and outspoken success of several transformative therapies, has intensified measures to seek cures (Mechler et al., 2015).

Remedies for rare diseases largely depend on the armory of logic provided by biochemical genetics and molecular cell biology. Specific therapies are available for several ultra-rare lysosomal disorders with strong genetic determinants. Effective treatment represents an innovative route to investigate complex pathophysiology–a strategy that could apply to illnesses in which mental health is deranged. This concept is not inimical to research discovery, even in the days when nearly all treatments were empirical. Once the exact mode of action of drugs was known, pathogenesis was better understood and with this came improved disease management – aspirin provides a simple example (Cheng et al., 2002).

Here a single lysosomal disease is the focus of an unconventional proposal to explore this retrospective approach to understand psychosis – a lasting frontier on the boundary of molecular neuroscience. The concept is simple: (1) Cerebral activity in health or mental illness is the product of the brain as a dynamic chemical laboratory driven by sensory input; small-effect gene variants may be important but their influence so far appears to be unfathomable. (2) Rare conditions due to unitary determinants with large effects provide opportunities for decisive scientific exploration. (3) Treatments that engage defined molecular targets in diseases with disturbed sanity are an opportunity to examine the biological basis of mental health.

### Psychiatric Manifestations of Metabolic Diseases

Psychiatric disturbances are a presenting feature of some inborn errors of metabolism but too often, identification of the cause is delayed until long after the systemic manifestations appear. With the move to early specialization and abbreviated exposure to general and neuropsychiatric training, the risk of misdiagnosis in a patient with a metabolic disease remains. When this occurs, not only is specific treatment directed to the metabolic defect denied, but inappropriate, even dangerous, medication can easily be given in error. Clearly, any salutary intervention is likely to be more effective before a permanent derangement due to neuro-metabolic injury occurs. Since lost opportunities for prompt treatment have dire consequences for the patient, their family, carers and society as a whole, awareness of unusual psychiatric manifestations or subtle clinical signs indicating an inborn metabolic disease is of crucial importance.

Table 1 illustrates the range of metabolic diseases associated with either acute or sustained psychiatric manifestations but is not a definitive list. A history of heredo-familial disease or overt systemic features with or without frank cognitive or neurological signs can expedite diagnosis but delays are the rule when psychiatric manifestations occur alone. There are several operational groups (Walterfang et al., 2013):-

**TABLE 1:** Inborn errors of metabolism with psychiatric features.

Disease group	OMIM No.	Psychiatric manifestations
**Cofactors and Small Metabolites**		
Wilson disease	277900	Labile and irritable, antisocial behavior, wide-ranging, obsessive-compulsiveness, psychosis
Cobalamin deficiency (CblC, or G)	277400	Disturbed behavior, visual hallucinations
Folate deficiency (e.g., of methylene tetrahydrofolate reductase)	236250	As above but catatonia reported
Lesch-Nyhan syndrome	300322	Dementia, aggression, self-mutilation
Porphyria (e.g., acute intermittent)	176000	Delusion, anxiety, mania, psychosis
Ornithine transcarbamylase deficiency (other genetic hyperammonaemias)	311250	Episodic alienation, catatonia, delusions, psychotic outbursts; protein aversion
Succinic semialdehyde dehydrogenase deficiency	234500	Attention deficit, hyperactivity, sleep disturbance, obsessive-compulsions and autism
Monoamine oxidase deficiency	309850	Dementia, violence and (sexual) deviancy
Non-ketotic hyperglycinaemia	605899	Coma, agitation, behavioral difficulties
Cerebrotendinous xanthomatosis	213700	Mood changes, hallucinations
**Peroxisomal Disorders**		
Adenoleukodsytrophy	300100	Attention deficit and disturbed behavior, fronto-temporal dementia mimics psychosis
**Lysosomal disorders**		
Sanfilippo disease subtypes A, B, C and D Mucopolysaccharidosis (MPS type 3 A–D)	252900, 252920, 252930 and 252940	Hyperactivity, cognitive decline and aggression Delayed systemic or skeletal MPS features
Hunter disease (MPS type 2)	309900	Cognitive decline, hyperactivity and aggression
Alpha-mannosidosis and β-mannosidosis	248500,248510	Confusion, hallucinations and psychotic episodes
Niemann-Pick disease type C1 & C2	257220,607625	Behavioral changes, attention-deficit hyperactivity disorder in juveniles; autistic and psychotic behavior in adults
Anderson-Fabry disease	301500	Depression, mild cognitive impairment
Metachromatic leukodystrophy	250100	Attention deficit and disturbed behavior, fronto-temporal dementia mimics psychosis
GM2 gangliosidosis (Tay-Sachs and Sandhoff diseases)	272800 268800	Depression, delusions, hallucinations, ideas of reference–schizophrenia may antedate neurological in ~50% adults

*Representative disorders in which psychiatric manifestations occur: individual diseases are grouped operationally according to the principal biochemical abnormality. OMIM refers to assigned numbers in Online Mendelian Inheritance in Man (http://www.ncbi.nlm.nih.gov/omim/).*

(I) Diseases that present with acute and recurrent attacks of confusion, sometimes mistaken for acute psychosis (e.g., urea cycle and homocysteine remethylation defects, hereditary tyrosinaemia type 1 and acute porphyrias). These episodes often result from periodic metabolic decompensation and overproduction of toxic metabolites. Systemic, as well as neurological signs usually develop during the attack.
(II) Diseases with long-standing psychiatric features that arise in adolescence or adulthood: catatonia, visual hallucinations–often aggravated by empirical interventions. Homocystinurias, Wilson disease, adrenoleukodystrophy, metachromatic leukodystrophy and some lysosomal disorders fall into this category. Personality and behavioral changes with delusions and altered mood give rise to non-diagnostic psychiatric features; schizophrenia is often suspected until cognitive decline or systemic disease appear.
(III) Metabolic errors without overt cognitive loss that attract immediate psychiatric attention – this includes patients with Wilson disease, homocystinurias, cerebrotendinous xanthomatosis, non-ketotic hyperglycinaemia, monoamine oxidase A deficiency. Lysosomal diseases are also included: the mannosidoses, mucopolysaccharidosis types 3 A-D, metachromatic leukodystrophy and GM2 gangliosidosis.

While declining cognition is almost inevitable in metabolic errors that affect the brain, several have psychiatric manifestations only and loss of cognition is either absent or extremely subtle. Some patients function persistently at a high level for years, even many decades, before mental capacity is affected.

Of the lysosomal diseases that fall into this category, metachromatic leukodytrophy and late-onset Tay-Sachs disease (GM2 gangliosidosis) are the best known. In metachromatic leukodystrophy, psychotic features accompany injury to sub-cortical white matter and fronto-temporal dementia. Behavior is disorganized and emotional responses are inappropriate; careful examination reveals short-term memory loss and confabulation in a manner resembling Korsakoff syndrome. The focus of this article is late-onset Tay-Sachs disease, in which at least half the patients have severe psychiatric disease.

### “The Most Diversified Chemical Laboratory of the Animal Economy” (Thudichum, 1884)

In 1884, JLF Thudichum published *A Treatise on the Chemical Constitution of the Brain* (Thudichum, 1884). A towering genius and practicing doctor, he had laboriously purified and characterized 140 distinct chemicals from extracts of bovine and human brains. Among these, Thudichum named an unusual component, an alkaloidal nitrogenous base and amino-alcohol, sphingosin(e) – “in commemoration of the many enigmas it presented to the inquirer.” Related constituents, e.g., sphingomyelin, are members of a vast array of sphingolipids distributed throughout nature. The brain has a rich content of sphingolipids: Tay-Sachs disease is one of 15 or so sphingolipidoses in which their lysosomal recycling is impaired.

### Tay-Sachs Disease (Desnick and Kaback, 2001) – And References Therein

Like Johann Thudichum, Waren Tay worked in London. In Whitechapel, 1881 Tay reported the first of several cases of what later was termed Tay-Sachs disease. Aged 12 months, the child had hypotonia and weakness of the neck and limbs after the first few weeks of age without paralysis: on ophthalmoscopy Tay, noted perifoveal pallor – the pathognomic retinal “cherry-red spot.” By 16½ months, the little girl was helpless and passive; optic atrophy was established (Tay, 1881). Without genetic screening, the autosomal recessive trait, Tay-Sachs disease occurs in ∼1 in 3500 newborn Ashkenazi Jews; at least 25 patients had been reported before Thudichum died in 1901. Bernard Sachs, a neurologist in New York, noted the disease in Ashkenazi families and conducted numerous neuropathological studies (Sachs and Strauss, 1910). There was no attempt to characterize the pathological lipid observed in microscopically neurons throughout the body. Despite intensive work, Thudichum’s rigorous chemical approach was either unknown or ignored and unfortunately he was not well-placed to pursue the powerful weapon of the clinical investigator – the study of exceptions. It was only in 1942 that Ernst Klenck identified gangliosides and the abnormality of GM2 in Tay-Sachs disease (Desnick and Kaback, 2001).

**FIGURE 1:**
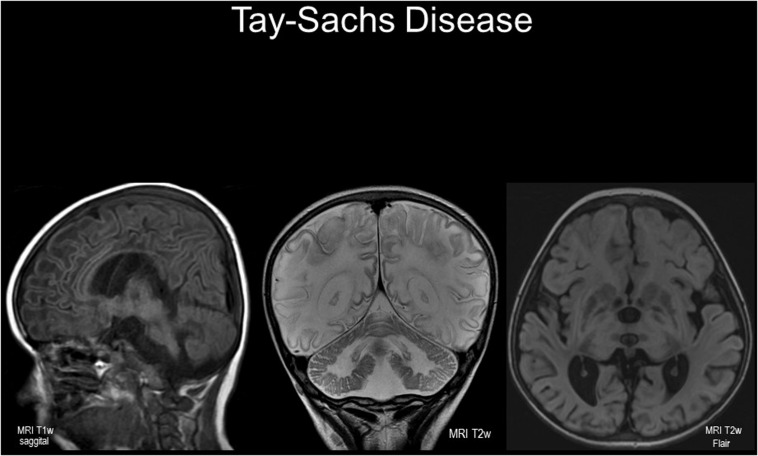
Cranial imaging in a 5 year-old child with infantile-onset GM2 gangliosidosis. Magnetic resonance images (left to right: sagittal T1w; coronal T2w; axial T2w FLAIR). Note the cerebral cortical and cerebellar atrophy with wide sulci and shrunken gyri; there are symmetrical diffuse white matter changes with reduced T1w intensity and hyper-intense signals in T2w images – the latter indicating demyelination. Abnormal signal intensity is seen in basal ganglia, peducles, and brain stem. Female infant born after normal delivery to unrelated white British parents without Ashkenazi ancestry; hypotonia noted at 7 months with cherry-red spots observed. Tay-Sachs disease confirmed enzymatically and by molecular analysis of the *HEXA* gene (well-known “severe” mutation frequent in British “Celtic” patients was identified). With scrupulous care and potentially salutary effects of a substrate-reducing agent (miglustat), the illness followed an unexpectedly protracted course. Nonetheless, characteristic startle responses to sounds, loss of motor skills, epilepsy and episodes of aspiration pneumonia led to deterioration with a peaceful death, aged 8 years.

**FIGURE 2:**
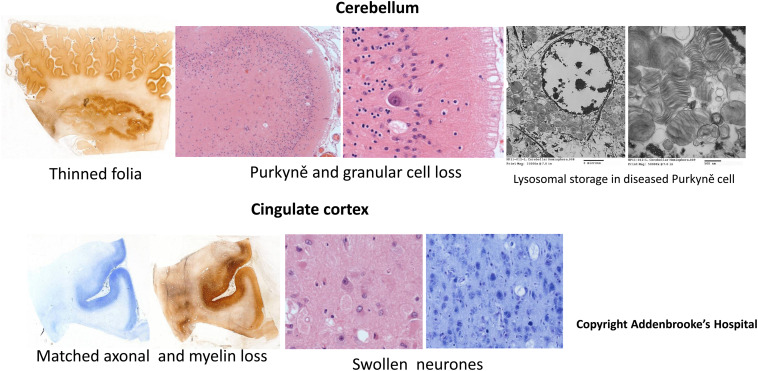
Neuropathological findings in a 3 year-old patient with GM2 gangliosidosis. Light microscopic images of brain sections from a non-Jewish British infant with biochemically confirmed classical infantile-onset Tay-Sachs disease showing, left to right, views of cerebellum (upper panels) and cerebrum, cingulate lobe, (lower panels) with increasing magnification revealing structural neuronal disease and cell loss as indicated with “ghosts.” Extreme right hand panels show ultrastructural features including florid lysosomal GM2 ganglioside storage pathology in a cerebellar Purkyně cell at different magnifications (bars indicate, respectively 2 μm and 500 nm). Macrocephaly with astrogliosis was present with striking cerebellar atrophy (total brain mass 1384 g, cerebellum and brain stem 113 g). Note special stains indicate the neuronal loss and disease in cerebellum and cerebral cortex associated with demyelination and axonal loss in subcortical white matter. (Courtesy Dr. Andrew Deane, Department of Pathology, Cambridge University Hospitals).

Figures 1, 2 extensive neurological injury involving nearly every neuron in a florid pathological process: at least 10% of the dry weight of the enlarged brain of infants dying of Tay-Sachs disease is due to the sialylated sphingolipid, GM2 ganglioside (normal, < 0.1%).

### The Biochemistry and Clinical Phenotype of Infantile Tay-Sachs Disease (Desnick and Kaback, 2001)

Tay-Sachs and the cognate Sandhoff disease, are due to deficiencies of one or both lysosomal acid hydrolases (EC 3.2.1.52–beta-N-acetylhexosaminidase) known, respectively, as the hexosaminidase A and B isozymes; identification of the enzyme defects was inferred from GM2 ganglioside structure. Mutations in *HEXA*, encoding the α-subunit of heteromeric isozyme A or HEXB encoding the common β-subunit of both isozymes, impair lysosomal recycling of GM2 ganglioside by hexosaminidase A activity–an activity also dependent on GM2 activator protein (itself rarely affected by mutations in the cognate *GM2A* gene).

Affected infants have axial hypotonia in the early months of life. Lack of visual fixation partly due to impaired visual cortical failure, and swallowing occur. There is loss of sight, speech and other faculties: even today, the baby is likely to die with seizures or aspiration pneumonia within 4 years. In this disease, an infant who does not sit up by 6 months of age, will never sit unaided. Sandhoff infants have an almost identical disease of the nervous system and modest systemic manifestations due to loss of hexosaminidase B activity toward glycosaminoglycans and glycoproteins.

### Attenuated Variants of GM2 Gangliosidosis – Tay-Sachs Disease (Rapin et al., 1976; Johnson, 1981; Harding et al., 1987; MacQueen et al., 1988; Desnick and Kaback, 2001; Frey et al., 2005; Neudorfer et al., 2005)

The most common GM2 gangliosidosis variant is infantile but in the attenuated spectrum, juvenile and adult subtypes are recognized. Juvenile disease typically presents at about 4 years of age with cerebellar ataxia; dementia with loss of speech and swallowing cause death due to aspiration or intractable seizures by 20 years.

Late-onset or adult Tay-Sachs and Sandhoff diseases are the paradigmatic focus of this *Perspective*. The former is the best documented but the author has clinical experience of both subtypes; when reviewed, one patient was 71 years old (Harding et al., 1987). The disease has protean manifestations and an expanding phenotype characteristically misdiagnosed by neurologists and psychiatrists alike. There is progressive impairment of gait as well as articulation in adolescence or late childhood; while unequivocally deficient, assays that utilize artificial fluorogenic substrates give plasma hexosaminidase A determinations 1–10% of mean reference values (inactivating mutations in classical Tay-Sachs disease are usually associated with < 1%). First, reported in Ashkenazi Jews, who harbor a widespread missense *HEXA* mutation, p.Gly269Ser, late-onset Tay-Sachs disease occurs in non-Jews, and p.Gly269Ser is also found; other HEXA variants occur and should be sought given that expressivity in affected pedigrees in variable.

A mimic of innumerable chronic neurological conditions, adult GM2 gangliosidosis frequently presents with anterior motor neuron weakness and wasting affecting selected muscle groups; ataxia and other cerebellar signs are variable. However, dystonia (sometimes with tremor complicated by chorea), dyskinesias, as well as neuropathy with mainly sensory or autonomic features occur. Inevitably, inappropriate clinical diagnoses of Friedreich’s ataxia, amyotrophic lateral sclerosis and other motor neuron diseases with or without peripheral neuropathy may arise. Dementia is variable and may be absent for many decades however its onset can be heralded by rapid dysarthria and facial grimacing. Abnormal ocular gaze, optokinetic nystagmus and supranuclear palsy are reported (Harding et al., 1987). Late-onset GM2 gangliosidosis should be considered in patients with multisystem neurodegenerative disorders, including those with adult onset. Even without an approved treatment, missed diagnosis is usually catastrophic because of the lost opportunities to counsel first-degree relatives.

### Psychiatric Manifestations of GM2 Gangliosidosis

Florid psychiatric manifestations occur in at least half the patients and may be the presenting feature. Mental aberrations occur in late-onset Sandhoff disease but in the author’s experience, these tend to be less marked than those in hexosaminidse A deficiency alone.

The most striking mental abnormalities are indistinguishable from classical schizophrenia and usually appear during puberty or in late adolescence (Rosebush et al., 1995). Often intellect and cognitive power are preserved (provided they are not confounded by intrusive delusions) (MacQueen et al., 1988; Frey et al., 2005).

Several patterns of this cruel neuropsychiatric disorder occur but classical hebephrenic schizophrenia with bizarre behavior, severe personality disintegration, erratic speech, agitation and childish quirks is well recognized. Disorganized thinking, delusions with ideas of reference and visual and/or auditory hallucinations, accompanied by inappropriate affect, are distressing. Recovery may occur but recurrent psychotic attacks erode the personality and induce severe depression. Paranoid schizophrenia, sometimes with violent reactions, also occurs. Neurological features may be completely absent for many years, although often aggravated by prescription drugs – particularly neuroleptics is psychiatric use (see below). Slurred speech, ataxia or cognitive impairment may emerge but frank dementia is unusual; the speech defect often accompanies impaired swallowing and the risk of aspiration pneumonia.

### Importance of Drug-Interactions in Neuronopathic Lysosomal Diseases (Johnson, 1981; MacQueen et al., 1988; Rosebush et al., 1995; Shayman and Abe, 2013)

Patients afflicted by the late-onset forms of GM2 gangliosidosis may show clinical deterioration when treated with psychotropic agents (tricyclic antidepressants, anticonvulsants, sodium valproate, and many antipsychotic drugs). Many of these drugs are strong cationic amphiphiles with classical lysosomotropic properties on which their psycho-pharmacological effects depend–the lysosomal proton gradient collapses, thus inducing phospholipidosis (Shayman and Abe, 2013). These agents exacerbate the neurological illness and wherever possible, they should be avoided (MacQueen et al., 1988; Rosebush et al., 1995).

### Therapeutic Opportunities in GM2 Gangliosidosis

Sachs referred to “this gravest and most fatal of all family diseases” (Sachs and Strauss, 1910), and GM2 gangliosidosis remains a paradigm of unmet need and opportunity at the boundary of biopharmaceutical developments in lysosomal diseases that include gene therapy (Mechler et al., 2015; Cachon-Gonzalez et al., 2018; Platt et al., 2019). However, for attenuated variants, innovation is based on clinical proof-of-concept in the related sphingolipid disorder, Gaucher disease, in which recycling of glucosylceramide is impaired. Here, substrate-reduction therapy is predicated on inhibitors of UDP-glucose: N-acylsphingosine D-glucosyltransferase, which catalyzes the biosynthesis of β-D-glucosylceramide – precursor of gangliosides and cognate glycosphingolipids (Merrill, 2011; Shayman and Larsen, 2014).

Eliglustat, a potent and highly selective inhibitor (IC_50_ ≈25 nM) now globally approved (as Cerdelga^®^), is an effective, first-line drug in Gaucher disease (Mistry et al., 2018). However, eliglustat cannot traverse the blood-brain barrier and is thus unsuitable for neuronopathic forms of Gaucher disease – or indeed GM2 gangliosidosis. Venglustat, an eliglustat analog, is able to enter the brain. At the time of writing, satisfactory completion of clinical 1-year phase 1/2 studies in neuronopathic, Gaucher disease has led to an extension phase (Schiffmann et al., 2020).

GM1, the parent ganglioside of GM2, as well as lactosylceramide and globosides are derived from glucosylceramide by the ordered action of glycosyltransferases on the luminal side of the trans-Golgi network after transmembrane “flipping” (Merrill, 2011). Thus the UDP-glucose: N-acylsphingosine D-glucosyltransferase (cytosolic face) is a common reaction target in Tay-Sachs/Sandhoff, Fabry and Gaucher diseases. The therapeutic potential of venglustat as a brain-penetrant inhibitor of glycosphingolipid biosynthesis, immediately lends itself to exploration in late-onset Tay-Sachs/Sandhoff diseases – a view supported by strong preclinical findings in the genetically coherent, Sandhoff strain mouse, with GM2 gangliosidosis: survival was increased, neuro-inflammation and indicative biomarkers (including brain gangliosides), were diminished (Marshall et al., 2019). These and other safety and efficacy findings has prompted further clinical exploration of venglustat (e.g., GBA-1-related Parkinson and Fabry disease). In adults with late-onset GM2 gangliosidosis, venglustat should decrease biosynthesis of β-D-glucosylceramide (and cognate gangliosides), thereby rebalancing glycosphingolipid salvage and breakdown. A trial to determine the efficacy, pharmacodynamics, pharmacokinetics, and safety is imminent (ClinicalTrials.gov Identifier: NCT04221450).

Clinical and biochemical studies (including CSF analysis) will confirm therapeutic target engagement and provide an opportunity for careful exploration of mental phenomena. Should there be effects on the psychotic and neuropsychiatric features of GM2 gangliosidosis, opportune clinical studies may yield deeper understanding of the biological basis of mental derangement. Further research should also address the molecular dynamics of sphingolipids such as GM1, GM2, GD2, and GD1a gangliosides in the brain and their relationship to activity-dependent synaptic strengthening and remodeling during development and memory formation (Olsen and Færgeman, 2017).

## Discussion

Organic disease has been reported to account for > 5% of patients who present with a first episode of psychosis (Johnstone et al., 1987). Most of the metabolic conditions are set out in Table 1 but perhaps the most alarming are those that present with features resembling schizophrenia. In these, beyond the dire effects of incarceration, neuroleptic drugs are likely to exacerbate the lysosomal disturbance and with that perpetuate the mental disturbance.

The extent to which mutant *HEXA* alleles or other genes implicated in lysosomal diseases are responsible for psychiatric disease in the general population is unknown. Initial genome-wide association studies failed to detect the strong genetic linkage between *GBA1* variants in Parkinson disease and Lewy body dementia – but the direct association with Gaucher disease led to decisive studies based on detection of numerous disease-related mutant alleles by sequencing. In other neurodegenerative diseases, there is the strong association of heterozygous variants of *PGN*, the progranulin gene with fronto-temporal dementia and variants of *GNPTAB, GNPTG*, and the functionally related *NAGPA* gene with non-syndromic stuttering. Since homozygous or biallelic mutations of *PGN* are responsible respectively for neuronal ceroid lipofuscinosis type 11 and for the mucolipidosis subtypes, characterized by multiple lysosomal effects, dose effects of heterozygous gene variants in causation of diverse neuropsychiatric diseases is clearly established (Huin et al., 2020; Raza et al., 2016). The extent to which such rare variants directly account for mental illness is emerging from population-wide whole-genome sequencing data – a promising approach in national health initiatives to improve diagnosis (Walkley et al., 2010).

Among the lysosomal diseases, aberrant mental behavior also occurs in Niemann-Pick C, MPS 2 and MPS 3 (A–D) – the latter caused by defective heparan sulfate breakdown: in all, ganglioside catabolism of neuronal cell membrane is impaired and secondary accumulation of gangliosides occurs. Where the lysosomal system is deranged, ectopic dendritogenesis, with bulbous axonal swellings (spheroids) principally in terminal trees are found. Electron microscopy shows amassed membrane structures and multivesicular bodies in association with axonal outgrowth meganeurites: these appear to alter interneuronal connectivity and occur preferentially in the cerebellum and thalamus, the most affected in GM2 gangliosidosis (Turro et al., 2020). Neuronal membranes are highly enriched in gangliosides, especially GM1 and others derived from the precursor glucosylceramide. As yet, the biophysical constraints exerted in their cognate lipid microdomains, which participate in the regulation of dendritic spine dynamics and hence memory formation, are not understood. However relevant to the thesis here, selective deletion of UDP-glucose: N-acylsphingosine D-glucosyltransferase in subsets of adult forebrain neurons has been shown to improve spatial memory and overcome loss of dendritic spines in the hippocampus of 5x familial AD mice, which model Alzheimer disease (Herzer et al., 2018). This tantalizing study is but one of many showing the central role of gangliosides in synaptogenesis and a proof of concept for the potentially salutary action of venglustat in GM2 gangliosidosis.

In the penultimate paragraph of his masterpiece written nearly 150 years ago (Thudichum, 1884), Thudichum, discoverer of sphingolipids and unwitting founder of the school of biological psychiatry, explicitly stated: “great diseases of the brain and spine, such as general paralysis, acute and chronic mania, melancholy, and others, will all be shown to be connected with specific chemical changes in neuroplasm….” Lysosomal diseases with disturbed ganglioside metabolism with challenging effects on mental health, evoke this classical work. Clinical investigation of novel brain-penetrant inhibitors of glycosphingolipid biosynthesis offers a direct way to test Thudichum’s assertion.

## Ethics Statement

Ethical review and approval was not required for study on human participants in accordance with the local legislation and institutional requirements. Written informed consent for participation was not required for the hypothetical ideas proposed in accordance with the national legislation and institutional requirements.

## Author Contributions

The author explored potential therapeutic opportunities prompted by clinical experience, experimental findings, and clinical research conducted in his laboratory and clinic with associates in third-party-sponsored clinical trials of substrate-reduction therapy for sphingolipid diseases.

## Conflict of Interest

TC was a Principal Investigator in clinical trials of eliglustat and currently venglustat in patients with sphingolipidoses (no personal remuneration) for Sanofi Genzyme.
